# Ledipasvir and Sofosbuvir in the Treatment of Early Hepatitis C Virus Infection in HIV-Infected Men

**DOI:** 10.1093/ofid/ofy238

**Published:** 2018-09-21

**Authors:** Paari M Palaniswami, Ahmed El Sayed, Benjamin Asriel, Jesse R Carollo, Daniel S Fierer, the New York Acute Hepatitis C Surveillance Network, Bisher Akil, Juan Bailey, Paul Bellman, Daniel Bowers, Krisczar Bungay, Susanne Burger, Aviva Cantor, Rachel Chasan, Robert Chavez, Rita Chow, Robert Cohen, Patrick Dalton, John Dellosso, Stephen Dillon, Eileen Donlon, Terry Farrow, Jose Fefer, Michael Gaisa, Rodolfo Guadron, Stuart Haber, Susan Hefron, Lawrence Higgins, Lawrence Hitzeman, Ricky Hsu, Shirish Huprikar, Victor Inada, Sneha Jacob, Livette Johnson, Barbara Johnston, Donald Kaminsky, Oscar Klein, Jeffrey Kwong, Jose Lares-Guia, Eric Leach, Randy Levine, Irina Linetskaya, Larisa Litvinova, Amisha Malhotra, William Mandell, Martin Markowitz, Gal Mayer, Eddie Meraz, Erik Mortensen, Joseph Olivieri, Charles Paolino, Punyadech Photangtham, George Psevdos, Asa Radix, Steven Rapaport, Roona Ray, Gabriela Rodriguez-Caprio, William Shay, Nirupama Somasundaram, Lembitu Sorra, Richie Tran, Antonio Urbina, Rona Vail, Francis Wallach, Wen Wang, Susan Weiss, Melissa Wiener

**Affiliations:** Division of Infectious Diseases, Department of Medicine, Icahn School of Medicine at Mount Sinai, New York, New York

**Keywords:** acute HCV, enhanced treatment responsiveness, HIV-infected men who have sex with men (MSM), sexualized methamphetamine drug use

## Abstract

**Background:**

Treatment of HIV-infected men during early hepatitis C virus (HCV) infection with interferon results in a higher cure rate with a shorter duration of treatment than during chronic HCV infection. We recently demonstrated that this phenomenon applied to interferon-free treatment as well, curing most participants with short-course sofosbuvir and ribavirin. Due to the significantly higher potency of the ledipasvir/sofosbuvir (LDV/SOF) combination, we hypothesized that we would be more successful in curing early HCV infections using a shorter course of LDV/SOF than that used for treating chronic HCV infections.

**Methods:**

We performed a prospective, open-label, consecutive case series study of 8 weeks of LDV/SOF in HIV-infected men with early genotype 1 HCV infection. The primary end point was aviremia at least 12 weeks after completion of treatment.

**Results:**

We treated 25 HIV-infected men with early sexually acquired HCV infection with 8 weeks of LDV/SOF, and all 25 (100%) were cured. Twelve (48%) reported sexualized drug use with methamphetamine.

**Conclusions:**

Eight weeks of LDV/SOF cured all 25 HIV-infected men with early HCV infection, including those who were actively using drugs. Based on these results, we recommend treatment of newly HCV-infected men during early infection, regardless of drug use, to both take advantage of this 8-week treatment and to decrease further HCV transmission among this group of men.

Early-stage hepatitis C virus (HCV) infection, commonly referred to as “acute” infection, is more interferon (IFN)-responsive than chronic HCV, enabling cure of up to 98% of patients with early HCV in just half the duration used to treat chronic HCV [1]. Many investigators of the international epidemic of sexually transmitted HCV infection among HIV-infected men who have sex with men (MSM) subsequently showed that early HCV infection in these men was also more IFN-sensitive than chronic HCV, curing about two-thirds with 6 months of IFN treatment, a much higher cure rate with only half the treatment duration compared with treatment of HIV-infected men with chronic HCV (summarized in [2]). With the availability of the first direct-acting antivirals (DAAs) against HCV, we extended these findings by adding telaprevir to IFN to significantly shorten treatment, down to 12 weeks, while significantly improving the cure rate [3, 4]. Others using these first-generation DAAs in addition to IFN subsequently published similar findings of successfully shortening duration of treatment [5, 6]. Based on these studies, we hypothesized that this substantial advantage in treating early HCV compared with chronic HCV was an inherent characteristic of early HCV infection, albeit insufficiently defined, which we have described as enhanced treatment responsiveness [2, 7]. The release of sofosbuvir (SOF), a potent HCV nucleotide inhibitor, allowed us to test this hypothesis. We used the IFN-free regimen of SOF+ribavirin (RBV) for 12 weeks, half the duration used for chronic HCV, curing 11 of 12 (92%) HIV-infected men with early HCV [8]. Two other studies that used this same regimen, 1 with a much shorter course, were less successful, however [9, 10]. Due to the small sizes of these studies and because they did not report sufficient laboratory data to assess characteristics that might predict enhanced treatment responsiveness, we had limited ability to advance our understanding of this phenomenon.

With the subsequent availability of the more potent ledipasvir (LDV)/SOF combination, we hypothesized that we would be able to collect more clinical information to improve our understanding of the phenomenon of enhanced treatment responsiveness and gather data using shorter and therefore less expensive treatment that might contribute to efforts to eliminate HCV. We therefore performed a study of the treatment of early HCV infection in HIV-infected men using LDV/SOF for 8 weeks, which is two-thirds of the length of treatment compared with the 12-week course studied in clinical trials [11] and is Food and Drug Administration approved for HIV-infected patients with chronic HCV infection.

## METHODS

### Participants and Study Design

This study was a prospective, open-label, consecutive case series performed in a single clinical practice. HIV-infected MSM suspected to have newly acquired HCV infection were referred to a practice (D.S.F.) within the Mount Sinai Medical Center through a network of providers of HIV health care in the New York City area (New York Acute Hepatitis C Surveillance Network) established to study incident HCV infection among HIV-infected MSM [3, 8, 12–17]. Written informed consent was obtained with approval of the Icahn School of Medicine Institutional Review Board, in accordance with the Helsinki Declaration of 1975, as revised in 2000.

The specific eligibility criteria were (1) having HIV infection and (2) having early genotype 1 or 4 HCV infection. For primary HCV infection, the criteria for early HCV were detection of HCV viremia in the setting of either HCV Ab seroconversion within the prior 6 months or a 2.5-fold ALT increase compared with the prior ALT test within the last 6 months in the absence of other identified causes of liver injury. For HCV re-infection, there was an additional criterion of documentation of a period of at least 12 weeks of aviremia after either treatment-induced or spontaneous clearance. The clinical onset of HCV infection was defined as the date of the first documented ALT elevation, HCV Ab seroconversion, or HCV viremia, whichever came first.

Because spontaneous clearance in men in this epidemic is uncommon (reviewed in [7]), we attempted to initiate treatment with LDV/SOF as soon as practicable after the initial evaluation visit to minimize ongoing HCV transmission [2, 7]. Treatment was obtained as part of clinical care through each participant’s insurance or, in the case denial of treatment by the insurance company, through charitable foundations.

The regimen was LDV/SOF taken daily, dispensed by the pharmacy to the participants in bottles of 28 tablets, with all 56 tablets taken, regardless of how long that took. Study visits were at treatment baseline (the day treatment started), weeks 4 and 8 of treatment, and weeks 4, 8, and 12 post-treatment. Adherence and side effects/adverse events were assessed by participant self-report at each study visit. The primary study end point was HCV VL <15 IU/mL at least 12 weeks after taking the last dose of LDV/SOF.

There were no exclusions for concomitant chronic HBV infection, active cancer, low CD4 count, opportunistic infection, or for active drug or alcohol use. There were no requirements for receiving or stability of antiretroviral (ARV) medication or suppressed HIV VL (defined as HIV VL < 50 copies/mL). The only excluded ARV was tipranavir.

### Laboratory Analysis

The HCV antibody test we used was the third-generation HCV enzyme immunoassay, version 2.0 (Abbott Laboratories); the HCV VL test was COBAS AmpliPrep/COBAS TaqMan HCV Test (Roche Diagnostics), lower limit of quantification 15 IU/mL (1.2 log_10_ IU/mL); the HCV genotype test was a commercial assay (ARUP Laboratories) in which portions of both the core and NS5B regions of the HCV genome are sequenced. We determined IFNL3/4 (formerly IL28B) SNP-RS12979860 C and T alleles using an allele-specific primer extension assay measured with the Luminex LX 200 instrument (Molecular Pathology Laboratory, Mount Sinai Medical Center). The upper limit of normal (ULN) for ALT was defined as 35 U/L. The confidence interval (95%) of the cure rate was calculated with Prism 7.0 software, using the “exact method” of Clopper and Pearson.

## RESULTS

### Baseline Demographic and HIV Characteristics

We enrolled and treated 25 HIV-infected MSM with early HCV infection with an 8-week course of LDV/SOF at the Mount Sinai Medical Center between November 2014 and December 2016. The baseline HIV characteristics are shown in Table 1. Twenty-four men (96%) reported having had semen ejaculated into their rectums after unprotected anal intercourse, and 12 (48%) reported having used methamphetamine during sex, the 2 significant risk factors for sexual acquisition of HCV in New York City [13]. Seven (58%) of the 12 who used methamphetamine during sex had injected, but none shared injection equipment.

**Table 1.** Baseline Demographic Characteristics of 25 HIV-Infected Men Treated for Early Hepatitis C Infection With Ledipasvir and Sofosbuvir

Characteristic	Result
Median age (IQR), y	38 (30–47)
Ethnicity, No. (%)	
White	9 (36)
Black	8 (32)
Hispanic	7 (28)
Pacific Islander	1 (4)
Primary HCV, No. (%)	21 (84)
HCV reinfection, No. (%)	4 (16)
IFNL3/4, No. (%)	
CC	13 (52)
CT	8 (32)
TT	4 (16)
Median time since HIV diagnosis (IQR), y	11 (6–15)
ARV prescribed, No. (%)	24 (96)
HIV suppressed, No. (%)	21 (84)
Median CD4 count (IQR), cells/μL	620 (510–798)
Sexualized methamphetamine use, No. (%)	12 (48)
Jaundice, No. (%)	3 (12)
Genotype, No. (%)	
1a	23 (92)
1b	2 (8)
Median peak ALT (IQR), U/L	765 (218–935)
Median peak HCV VL (IQR), log_10_ IU/mL	6.2 (5.2–6.9)
HCV VL flux >1 log_10_ IU/mL, No. (%)	19 (76)
Median HCV VL flux (IQR), log_10_ IU/mL	1.9 (0.9–4.3)
Median time from HCV clinical diagnosis to start of treatment (IQR), wk	18 (10–27)
ALT at start of treatment (IQR), U/L	173 (61–396)
HCV VL at start of treatment (IQR), log_10_ IU/mL	5.1 (4.2–5.9)

Abbreviations: Ab, antibody; ALT, alanine aminotransferase; ARV, antiretroviral; CD4, cluster of differentiation 4; IQR, interquartile range; HCV, hepatitis C virus; IFNL3/4, interferon lambda 3/4; IU, international units; U, unit; U/L, units per liter; VL, viral load.

### HCV-Related Characteristics

**Figure 1.**
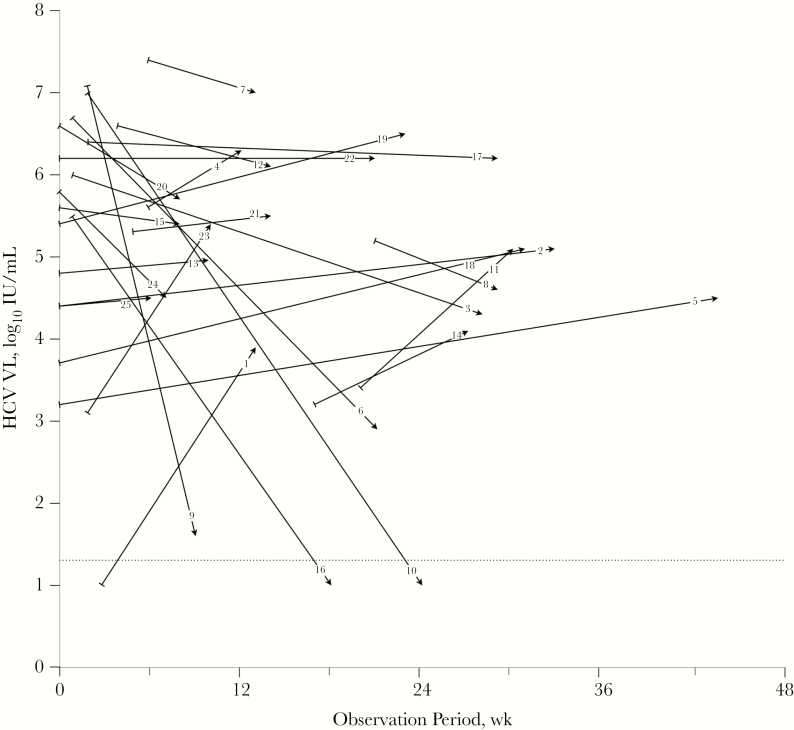
Trajectory of HCV VL from first measurement through initiation of treatment of 25 HIV-infected men with early hepatitis C infection. For each participant, the first-measured HCV VL (log_10_-transformed) is denoted by a right tack (⊢), and the HCV VL at treatment initiation is denoted by an arrowhead, with the points connected by a line, labeled corresponding to Table 2. The points are placed along the y-axis corresponding to the magnitude of the VL, and along the x-axis corresponding to the time after clinical onset of HCV infection that the VL was obtained. Most participants had their first HCV VL obtained after their date of clinical onset; hence most first points are to the right of the x-axis origin. The horizontal dotted line represents the lower limit of quantification of the HCV VL test. For the purposes of the figure, HCV VL below this threshold were assigned a value of 1.0 log_10_ IU/mL. Abbreviations: ALT, alanine aminotransferase; HCV, hepatitis C virus; VL, viral load.

Twenty-one (84%) men had primary HCV, and 4 had re-infection after earlier successful treatment or spontaneous clearance; none were treated twice with this protocol. Two men were symptomatic (both jaundiced) at the time of HCV diagnosis, and 1 other developed jaundice after diagnosis but before treatment; jaundice resolved in all 3 before the start of treatment. Five (21%) of the 21 men with primary HCV presented with negative HCV Ab; all seroconverted before treatment. The baseline HCV characteristics are shown in Table 1. The HCV VL declined by >1 log_10_ IU/mL between the first measurement after HCV diagnosis and treatment baseline in 6 (24%) men, increased by >1 log_10_ IU/mL in 7 (28%), and was within 1 log_10_ IU/mL in 13 (52%) (Table 2, Figure 1). The overall pattern of ALT fluctuation was parallel to the changes in HCV VL, although ALT tended to decline in those without large changes in HCV VL (Table 2). Examining more closely those at the extremes of endogenous virological control at the start of treatment, 6 (25%) men had VLs of >6 log_10_ IU/mL, and 2 men had VL fluctuations down to <1.2 log_10_ IU/mL on the day of treatment initiation (Table 2, Figure 1). Finally, at treatment initiation, 15 had maintained endogenous suppression of viremia below 5.2 log_10_ IU/mL, that is, at least 1 log_10_ IU/mL below the HCV VL typical for chronic HCV in HIV-infected patients [18], and 8 of 10 who had VLs >5.2 log_10_ IU/mL had concomitant ALT levels >5-fold above ULN (Table 2).

**Table 2.** Paired Hepatitis C Viral Load and Alanine Aminotransferase Measurements of 25 HIV-Infected Men With Early Hepatitis C Infection Treated With Ledipasvir and Sofosbuvir

Participant	First Available Paired Measurements		Start of Treatment Paired Measurements		Time Between Measurements, wk
ALT, U/L	HCV VL, log_10_ IU/mL	ALT, U/L	HCV VL, log_10_ IU/mL
1	26	<1.2 ND	47	3.9	10
2	74	4.4	64	5.1	33
3	2099	6.0	66	4.3	27
4	166	5.6	101	6.3	6
5	42	3.2	72	4.5	44
6	3762	6.7	51	2.9	20
7	258	7.4	132	7.0	7
8	293	5.2	88	4.6	8
9	824	7.1	33	1.6	7
10	767	7.0	17	<1.2 ND	22
11	53	3.4	173	5.1	10
12	389	6.6	252	6.1	10
13	1097	4.8	253	5.0	10
14	170	3.2	235	4.1	10
15	823	5.6	332	5.4	8
16	765	5.5	40	<1.2 D	17
17	840	6.4	395	6.2	27
18	2185	3.7	334	5.1	31
19	133	5.4	450	6.5	23
20	310	6.6	397	5.7	8
21	595	5.3	545	5.5	9
22	206	6.2	767	6.2	21
23	161	3.1	749	5.4	8
24	50	5.8	904	4.5	7
25	80	4.4	58	4.5	6
Median (IQR)	293 (107–824)	5.5 (4.1–6.5)	173 (61–396)	5.1 (4.2–5.9)	10 (8–23)

Abbreviations: ALT, alanine aminotransferase; D, detected; IU, international units; ND, not detected; U, unit; U/L, units per liter; VL, viral load.

### Treatment

Our protocol was to treat all men as soon as practicable after diagnosis, but in this study, subject to the exigencies of clinical practice, the median time to treatment was 18 weeks from clinical diagnosis of HCV infection (Table 1); the longest time to treatment was 44 weeks after diagnosis (Figure 1). The main reasons for delays in treatment were both delays in diagnosis, usually delayed HCV VL testing (Figure 1), and delays in the clinical system, including late referrals and difficulty obtaining approval from public and private insurance companies or support for medication costs from charitable foundations.

The actual duration of treatment, that is, the time it took to take all 56 doses, was longer than 8 weeks in 6 (24%) participants, who took 9 weeks to complete their 56 doses, and shorter in 1, who finished all 56 doses in just 7 weeks. None were lost to follow-up or reinfected before assessment for clinical cure. All 25 men had HCV VLs <15 IU/mL at least 12 weeks after completing treatment.

### Safety

There were no treatment-related side effects other than minor headache and fatigue, the known side effects of LDV/SOF [19], reported by 9 (36%) men, including in the 1 man who took his 8-week course in 7 weeks. Twenty (80%) men were receiving ARV regimens containing tenofovir disoproxil fumerate (TDF). Twelve (48%) of the 20 taking TDF were also taking a cobicistat- or ritonavir-boosted ARV. The median creatinine at the start of HCV treatment in those taking TDF was 1.1 mg/dL, and it was unchanged at the end of treatment. There were no on- or post-treatment HIV-related events.

## DISCUSSION

In this study, we successfully cured early HCV in all 25 HIV-infected men with only 8 weeks of LDV/SOF. This result extends our previous work, in which we treated early HCV with short courses of less potent regimens and obtained cure rates comparable to or better than when those regimens were used to treat chronic HCV [3, 8], leading to our description of enhanced treatment responsiveness as a characteristic of early HCV infection [2, 7, 8].

Although our results suggest that 8 weeks of LDV/SOF is a promising treatment for early HCV in HIV-infected patients, with no treatment failures in this study, we were not able to further define the clinical characteristics associated with treatment failure. The lack of treatment failures, though, suggests that treatment can still be further shortened, but to do so, we will need to better understand the characteristics of enhanced treatment responsiveness of early HCV. We postulate that enhanced treatment responsiveness is the manifestation of the still-preserved host immune response acting synergistically with antiviral drugs and that we may be able to use clinical characteristics to identify the robustness of this response over time. One clinical characteristic reflecting the still-preserved host immune response is low VL, which has consistently been associated with treatment success. High VL, in the 7-log range, however, presents a less clear picture, as our observations (Daniel S. Fierer, MD, unpublished data, 2018) [3, 8] suggest that high VL itself is not a sufficiently accurate indicator of cure by short-course treatment. To improve the estimation of enhanced treatment responsiveness, we have posited that high ALT levels (eg, >5-fold above ULN), as an indirect indicator of immune-based hepatocellular killing, be considered along with VL status to help guide the use of short-course therapy. For instance, a patient with early HCV and a high VL but a concomitant robust ALT elevation might still be within the period of enhanced treatment responsiveness, whereas a patient with the same duration of infection but a high VL and only a nominal concomitant ALT elevation might be too far into the transition to the chronic HCV phenotype to treat with short-course therapy.

The results of other studies of short-course treatment of early HCV appear to be overall consistent with these hypotheses. Among the 23 evaluable HIV-infected individuals with early HCV in the study of Rockstroh et al. [20], treatment with 6 weeks of LDV/SOF resulted in 3 (13%) virologic failures. All participants were reported to have been treated within 24 weeks of infection. The 3 who failed treatment all had HCV VLs close to 7 log_10_ IU/mL at the start of treatment, but ALT levels were not provided for those participants. The other study that used 6 weeks of LDV/SOF involved 20 HIV-uninfected participants; most presented symptomatically and were treated very quickly after diagnosis (median, 33 days). All were cured. Among the 4 participants in this study who had substantial upward fluctuations of VL from screening to start of treatment, including 1 to >7 log_10_ IU/mL, all had concomitant ALT elevations substantially greater than 5-fold above ULN at the time of treatment [21]. Two other studies in HIV-infected participants with early HCV assessed 8 weeks of treatment, both with complete success. Naggie et al. [22] cured all 27 participants using 8 weeks of LDV/SOF. The median time between diagnosis and treatment (15 weeks) was a little shorter than in our study, but the median VL (6.2 log_10_ IU/mL) was higher and the median ALT (133 U/L) lower than in our study. Martinello et al. [23] treated with 8 weeks of the no longer widely used regimen of paritaprevir/ritonavir/ombitasvir, dasabuvir, and ribavirin and cured all 23 HIV-infected participants. The median time between diagnosis and treatment (23 weeks) was the longest among these studies, although despite this longer time to treatment, the median HCV VL (5.7 log_10_ IU/mL) and ALT level (~150 U/L) were about intermediate between the Naggie et al. study and ours. Taken together with our study results, these latter 2 studies further support that 8 weeks of a potent-combination DAA regimen is effective in the treatment of early HCV, perhaps in particular in those whose enhanced treatment responsiveness period may have been waning, as judged by the criteria of higher VL and lower ALT. The 2 studies of the somewhat shorter treatment duration of 6 weeks suggest that careful selection of patients becomes more important as the treatment course becomes shorter. We speculate that better selection could be done by relying less on duration of infection, that is, de-emphasizing the 24-week estimated duration of infection as a cutoff for treatment for short-course therapy, and instead focusing more on clinical characteristics of the individual patient, such as evolution of VL and ALT levels.

We therefore suggest that future studies be performed to prospectively test these and similar hypotheses and include collection of more complete clinical information in parallel with immunological and virological studies to understand the correlates of enhanced treatment responsiveness. These data may allow us to create a better tool to predict the robustness of an individual’s enhanced treatment responsiveness to optimize both treatment duration and ultimately both improve clinical care and decrease cost of treatment if durations as short as 4 weeks prove to be effective enough.

We would like to note a few other observations stemming from our results. We have again confirmed that treatment of active methamphetamine users is highly effective, reinforcing that active drug users should be treated (reviewed in [24]). We also note that 100% adherence with this 8-week LDV/SOF regimen was not necessary to achieve 100% cure as long as all tablets were eventually taken. This study lends additional evidence that early treatment of HCV in HIV-infected MSM is safe and effective, which should prompt national guidelines committees [25] to recommend immediate treatment of newly infected individuals, which would have the added benefit of reducing the spread of HCV, consistent with the test-and-treat strategy used for HIV infection. Finally, many of the delays we encountered in getting our patients treated could have been prevented by eliminating the restrictive regulations imposed by third-party payers that are an obstacle to early treatment of HCV [26].

Although the treatment results of our study are promising, there are limitations to these data. The study was performed in a single clinical practice that is expert in diagnosing and treating early HCV infection, so our high cure rate might not be generalizable to other clinical settings. The recently completed multicenter study of Naggie et al. that we discussed used the same treatment regimen for the same duration and had the same result [22], however. Our study was small compared with studies of chronic HCV, resulting in confidence intervals of 86% to 100%, but it is comparable in size to the published studies of early treatment of HCV infection. Just over half of the men had the favorable IFNL3/4 CC genotype, higher than found in studies of chronic HCV, although this proportion is usual in cohorts of early HCV infection (as reviewed in [3]). Finally, although observational studies have supported the use of 8 weeks of LDV/SOF in the treatment of HIV-uninfected patients with chronic HCV, the lack of sufficient evidence for using 8 weeks to treat HIV-infected patients is reflected in the American Association for the Study of Liver Diseases/Infectious Diseases Society of America guidelines that specifically recommend against this practice, regardless of viral level [27].

In summary, we cured all 25 HIV-infected men from their early genotype 1 HCV infections using an 8-week short-course regimen of LDV/SOF. We continue to advocate that in clinical practice, HIV-infected men with early HCV should be treated immediately, including those with active drug use, to take advantage of short-course treatment for most and to use treatment as prevention of sexually transmitted HCV infection among MSM [28], which is necessary to meet the WHO goal of global HCV elimination.

## Notes

***Prior presentations: *** Poster 1996. American Association for the Study of Liver Disease; November 14, 2016; Boston MA. Poster THU-250. European Association for the Study of Liver Disease; April 20, 2017; Amsterdam, the Netherlands.
